# Patterns, determinants and barriers of health and social service utilization among young urban crack users in Brazil

**DOI:** 10.1186/1472-6963-13-536

**Published:** 2013-12-28

**Authors:** Marcelo Santos Cruz, Tarcisio Andrade, Francisco I Bastos, Erotildes Leal, Neilane Bertoni, Lara Lipman, Chantal Burnett, Benedikt Fischer

**Affiliations:** 1 Institute of Psychiatry, Federal University of Rio de Janeiro, Rio de Janeiro, Brazil; 2 Department of Community and Family Health, Federal University of Bahia, Salvador, Brazil; 3 Institute of Communication and Scientific Information & Technology for Health, Oswaldo Cruz Foundation, Rio de Janeiro, Brazil; 4 Centre for Applied Research in Mental Health and Addiction, Faculty of Health Sciences, Simon Fraser University, 2400 - 515 W Hasting St, Vancouver, BC V6B 5K3, Canada; 5 Social & Epidemiological Research, Centre for Addiction and Mental Health, Toronto, Canada

**Keywords:** Crack use, Health services, Treatment, Barriers, Brazil, Marginalized populations

## Abstract

**Background:**

Crack use is prevalent across the Americas, and specifically among marginalized urban street drug users in Brazil. Crack users commonly feature multiple physical and mental health problems, while low rates of and distinct barriers to help service use have been observed in these populations. This study examined profiles and determinants of social and health service utilization, and unmet service needs, in a two-city sample of young (18–24 years), marginalized crack users in Brazil.

**Methods:**

N = 160 study participants were recruited by community-based methods from impoverished neighborhoods in the cities of Rio de Janeiro (n = 81) and Salvador (n = 79). A mixed methods protocol was used. Participants’ drug use, health, and social and health service utilization characteristics were assessed by an anonymous interviewer-administered questionnaire completed in a community setting; descriptive statistics on variables of interest were computed. Service needs and barriers were further assessed by way of several focus groups with the study population; narrative data were qualitatively analyzed. The study protocol was approved by institutional ethics review boards; data were collected between November 2010 and June 2011.

**Results:**

The majority of the sample was male, without stable housing, and used other drugs (e.g., alcohol, marijuana). About half the sample reported physical and mental health problems, yet most had not received medical attention for these problems. Only small minorities had utilized locally available social or health services; utilization appeared to be influenced by sex, race and housing characteristics in both sites. Participants cited limited service resources, lack of needs-specific professional skills, bureaucratic barriers and stigma as obstacles to better service access. However, most respondents stated strong interest and need for general social, health and treatment services designed for the study population, for which various key features were emphasized as important.

**Conclusions:**

The study contributes substantive evidence to current discussions about the development and utilization of health and treatment interventions for crack use in Brazil. Based on our data, crack users’ social, service needs are largely unmet; these gaps appear to partly root in systemic barriers of access to existing services, while improved targeted service offers for the target population seem to be needed also.

## Background

Crack use is prevalent across the Americas. Since the early 1990s, crack use has widely spread in cities across Brazil, and has become prevalent among street drug users [1,2]. While no precise epidemiological assessments are currently available, the crack user population in Brazil has been estimated to be up to 1 million, largely concentrated in young, marginalized, urban populations [3-5]. The phenomenon of crack use has spawned extensive attention and controversial debates in Brazil about appropriate intervention strategies, also due to its extensive social impact, including extensive violence (e.g., gun violence) and concerns about community health and safety, especially in impoverished neighborhoods (‘cracolandias’) affected by this problem [6-8].

Drawing on both data from Brazil and other settings in the Americas (e.g., North American cities) where crack use is also common, it is evident that crack users typically feature extensive health and social problems, even when compared to other illicit drug users [9-11]. For example, most crack users – while typically young – are characterized by high degrees of socio-economic marginalization, i.e. are commonly underhoused or homeless, and root from economically disadvantaged or impoverished backgrounds [12-14]. Many crack users report extensive involvement in both property and violent crimes – largely related to their involvement in the drug trade – as well as with the criminal justice system (including common incarceration) [15-20]. While crack use in Brazil has largely replaced previously common forms of injection drug use (IDU), most crack users are active poly-drug users (e.g., involving alcohol, cannabis, cocaine, other stimulants) with high levels of related substance use disorders [1,9,21,22]. Finally, crack users commonly feature extensive mental and physical co-morbidities. For example, mood (e.g., depression), psychotic (e.g., schizophrenia) and personality disorders have been shown to be disproportionately prevalent among crack users [23-26]. Furthermore, both Blood-Borne Viruses (BBVs) (e.g., HIV and/or Hepatitis B/C Viruses) are disproportionately common among crack users – influenced by both extensive sexual (e.g., sex trade involvement or sex-for drug exchanges) and/or drug use related risk behaviors (e.g., drug use paraphernalia sharing) [27-32].

Based on the common profile of extensive social and health risks and problems, crack users consequentially are in high need of appropriate social and health services and interventions [33-35]. In Brazil, social and health services targeting drug users have been substantially expanded since 2000 [36]. Key elements of this strategic expansion are out-patient and multi-disciplinary psycho-social service centers for alcohol and drug users (Centro de Atencao Psicossocial Alcool e Drogas [CAPS-AD]) [37-39]. While their coverage is still considerably below targets, close to 300 CAPS-ADs had been newly implemented by 2011; these are supported by federal and municipal resources, and accessible free of charge by their users [39]. In addition, multiple other social and health services – provided either by public or non-governmental organizations – are available for drug users in large cities, typically including community health centers, social assistance centers, therapeutic communities and in-patient treatment services [40]. Overall, there has been a substantive rise (from 236,770 in 2006 to 281,720 in 2011) in out-patient consultations for drug users [36].

Evidence, however, suggests that only small proportions of drug users do access or receive the social or health (including treatment) services they need. For example, North American data suggest that only limited proportions of problematic drug users receive basic social or health services [41-43], and receive targeted interventions or services for key health risks, such as BBV/STD risks or problems, only in exceptional cases [44]. A heterogeneous variety of reasons or barriers for drug users not utilizing key services have been documented. Barriers reported for access to drug abuse treatment in Latin-American countries include: lack of treatment professionals, services or facilities; perceived stigma; excessive costs; insufficient treatment or medication options; long wait times or limited working and opening hours; geographic distance or lack of transportation options [45]. North American studies have shown the role of geographic locations of services [46,47] and the lack of confidence or trust of patients in the health system as key access barriers [48]. Not considering themselves ill or lack of motivation for treatment [49], and culturally inappropriate service contexts, are additional reasons for not accessing treatment [50]. Distinct service access barriers exist for women, for example as relating to the fear of losing custody for their children or negative repercussions in the context of care when pregnancy occurs [49,51]. In the US, ethnic and racial factors – reflecting socio-economic inequities – have been shown to influence screening and referrals for drug problems [52]; for example, Blacks and Hispanics have been shown to be less likely to receive treatment [53,54]. Similarly, in Brazil, marginalized or impoverished populations have been shown to experience disproportionate barriers to health care or addiction treatment services [37]. For street-involved drug users, bureaucratic barriers – related to the lack of proper identification or health cards – are reported as barriers for treatment access [54].

This multi-methods study examined aspects of social and health service utilization among a sample of young marginalized crack users in two Brazilian cities (Rio de Janeiro and Salvador). Specifically, the study aimed to: 1) describe the utilization of key social and health services and identify factors associated with service use based on quantitative data; and 2) examine experiences, factors and dynamics of service access and use on the basis of qualitative data.

## Methods

The study [for details see Cruz et al. [55]] relied on quantitative and qualitative data from a cross-sectional multi-site study of regular street-involved crack users recruited from impoverished neighborhoods in Rio de Janeiro (e.g., Jacarezinho and Manguinhos) and Salvador (e.g., Pelourinho, Calabar, Ribeira, Fazenda Coutos and Valéria) with known large crack user populations. Participant recruitment was facilitated by community-based contact persons (e.g., community workers) with direct access to the target population. The community contacts disseminated information about the study to potential participants, who were then assessed for study eligibility on the basis of a few short screening questions.

The study’s eligibility criteria included: 1) crack use on three days or more per week in the last three months; 2) 18 to 24 years of age; and 3) consent to participate in all study elements. Individuals either acutely intoxicated or experiencing acute mental health problem episodes, or displaying aggressive or other problematic behavior that would impede assessment were not included. If eligible, study participants were guided to the community-based local study offices (located at the Manguinhos Emergency Room unit in Rio, and at the Federal University of Bahia located in the center of Salvador) for assessments.

Individual assessments were conducted in a private space in the local study sites, following the participant’s informed written consent. Quantitative assessments consisted of an interviewer-administered questionnaire with 31 items on socio-demographic and drug use characteristics, and health and treatment service needs. A total of 175 (95 in Rio and 80 in Salvador) individuals were screened for study eligibility (14 were excluded for age, 1 for drug use criteria), and a total of n = 160 study assessments (81/79) were completed between November 2010 and June 2011. All data were sent to the Oswaldo Cruz Foundation (FIOCRUZ) for processing and analysis. Questionnaire data were scanned using Teleform® procedures and manually quality-checked; statistical analyses were performed using STATA v.9. Descriptive analysis for key social-demographic, health and drug use, and the main social, health and treatment service variables of interest were reported by site. Then, exploratory analyses of univariate associations between ‘any health or social service use in the past 30 days’ (collapsed omnibus variable) and select variables of interest (identified from existing evidence and experiences elsewhere) were conducted by site, including: Sex, age, ethnicity, housing status, education level, arrest history (past year), length of crack use (in months), sexual risk behavior (sex without condom in the last 30 days), current alcohol use (past 30 days), self-rated physical health, self-rated mental health, and oral sores, burns or wounds (last 30 days).

Qualitative data on utilization, barriers and needs related to social, health and service utilization were collected from a total of 12 focus groups (total number of distinct participants n = 44), 8 in Rio de Janeiro (n = 31) and 4 in Salvador (n = 13), with a numeric range of 5 to 8 participants per focus group, conducted in community-based settings between June 2011 and August 2011. There were several focus group participants who participated in more than one focus group in both sites, in order to allow to explore key issues in adequate depth, and hence included repeat participants. The focus groups were led by trained facilitators, following a semi-structured guide of key questions (see Appendix 1), which had been pilot-tested and adapted following results. The focus groups had a median duration of 30–45 minutes; their content was audio-taped and transcribed. The narrative data from the transcripts were manually coded and organized by the major emerging themes, organized and extracted through a systematic and reflective process [56], mainly focusing on practices of, barriers to and needs related to service utilization, also especially considering similarities and differences by site.

The study protocol was approved by the Ethical Review Committee, Institute of Psychiatry, Federal University of Rio de Janeiro as well as the Brazilian National Ethics Committee (CONEP 519/2010).

## Results

### Quantitative results

In terms of key socio-demographic characteristics, the mean age of the samples in both study sites was 21 years (range 18–24; Standard Deviation [SD] 2.2 and 2.1, respectively). The mean length of crack use was 46 months (Rio; SD: 35) and 55 months (Salvador; SD: 39), respectively. As displayed in Table 1, the respective majorities were male and of black or mixed race, and had incomplete high-school education. While in Rio, the majority was characterized by unstable housing and had not been arrested in the past year – the opposite was the case in Salvador. About 4 in 10 participants in Rio, and 7 in 10 in Salvador reported current income from paid formal or informal work. Participants had an average history of 4 (Rio) and 5 (Salvador) years of crack use. The majority in both sites reported current use of alcohol, tobacco, marijuana and cocaine.

**Table 1:** Key socio-demographic and drug use characteristics of sample, by site

	**Rio de Janeiro**		**Salvador**	
	**(n = 81)**		**(n = 79)**	
	**n**	**%**	**n**	**%**
**Sex**				
Male	54	67	70	89
**Color/race**				
White	8	10	5	6
Black	31	38	32	41
Mixed race or other	42	52	42	53
**Marital status**				
Single	57	70	56	71
**Education**				
Incomplete	69	86	62	79
Elementary				
Schooling or less				
**Housing status**				
Lives in own or family	19	24	58	73
House or apartment				
Unstable housing	61	76	21	27
(including homelessness)				
**Arrested by police (in the past year)**	23	28	44	56
**Paid work (legal or illegal)**	34	42	55	70
**Other drugs used (in last 30 days)**				
Alcohol	61	75	72	96
Tobacco	76	94	62	76
Cocaine	55	68	67	85
Marijuana	64	79	71	90

While half the sample reported good or better physical health status in Rio, a quarter did in Salvador (Table 2); just under half reported physical health problems in both sites, with most seen as related to drug use. While most respondents did not receive medical attention for their physical health problems, the majority expressed desire for medical attention. Just over half in Rio, and just over one third of participants in Salvador reported ‘good’ or better mental health; just over one third in Rio, and just over half in Salvador reported current mental health problems. Some 4 in 10 in Rio, and three quarters in Salvador reported their mental health problems related to their drug use. Most respondents reported that they did not receive medical attention for their mental health problems, though the majority expressed desire for such attention.

**Table 2:** Key health risks and status characteristics of sample, by site

	**Rio de Janeiro (n = 81)**		**Salvador (n = 79)**	
	**n**	**%**	**n**	**%**
Physical health status in past 30 days*				
Excellent, very good, or good	43	53	19	24
Fair or poor	38	47	54	68
**Had physical health problems in past 30 days**	32	40	36	46
Physical health problems related to drug use	23	72	26	72
Received medical attention for physical health problems	4	13	9	25
Did not but would have liked to receive medical attention for physical health problems	24	75	30	83
**Mental health status in past 30 days**				
Excellent, very good, or good	45	56	30	38
Fair or poor	35	44	44	56
Had mental health problems in past 30 days**	30	37	44	56
Mental health problems related to drug use	12	40	32	73
Received medical attention for mental health problems	0	0	2	5
Did not but would have liked to receive medical attention for mental health problems	17	57	32	73

*6 missing cases in Salvador; **1 missing case in Rio; 5 missing cases in Salvador.

Each of the available social or health services were only utilized by small minorities of the local samples (Table 3). In Rio, food banks (26%), shelters (12%) and community health centers (10%); in Salvador, community health centers (11%), food banks (6%) and shelters and hospitals (5% each) had been utilized by the largest proportions of participants, though each were only used on a minority of days in the past month. In Rio, virtually no participant reported any of the various service options they desired to use as unavailable or inaccessible; in Salvador, such constellations were reported by small minorities at most. In addition, substantive sample majorities in both sites (74% in Rio and 88% in Salvador; data not shown) indicated that they would utilize a special facility with services designed and targeted specifically for drug users if available.

**Table 3:** Social and health service utilization and needs in sample, by site

	**Rio de Janeiro (n = 81)**			**Salvador (n = 79)**		
	**Used service in the**	**Number**	**Not available or**	**Used service in the**	**Number of**	**Not available or**
	**last 30 days**	**of days**	**unable to access**	**last 30 days**	**days**	**unable to access**
	**n (%)**	**Mean (SD)**	**n (%)**	**n (%)**	**Mean (SD)**	**n (%)**
Shelter	10 (12)	7.8 (6.3)	0 (0)	4 (5)	11.0 (16.5)	4 (5)
Food bank	21 (26)	8.1 (7.6)	0 (0)	5 (7)	8.5 (14.3)	3 (4)
Community health center	8 (10)	5.6 (10.8)	0 (0)	9 (11)	2.7 (3.1)	8 (10)
Hospital or emergency room	1 (1)	1.0 (−)	1 (1)	4 (5)	5 (2.8)	0 0
Needle exchange or outreach program	2 (2)	1.0 (−)	0 (0)	1 (1)	*	2 (2)
Mental hospital	0 (0)	-	0 (0)	1 (1)	30 (−)	1 (1)
Drug abuse treatment service	0 (0)	-	0 (0)	0 (0)	-	4 (5)
Therapeutic community	0 (0)	-	1 (1)	2 (3)	11.5 (12.0)	1 (1)
Other	0 (0)	-	0 (0)	0 (0)	-	0 (0)
CAPS AD (Alcohol and Other Drugs Psychosocial Centre)	0 (0)	-	0 (0)	1 (1)	30 (−)	3 (4)
University drug abuse service	0 (0)	-	0 (0)	0 (0)	-	5 (6)
Other	0 (0)	-	0 (0)	1 (2)	2 (−)	0 (0)

*missing value.

**Figure 1:**
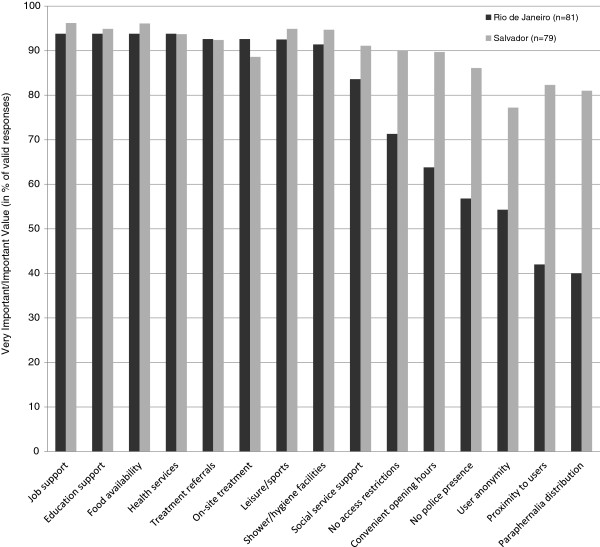
Factors influencing the potential utilization of social or health services in sample, by site.

The self-rating of specific service characteristics potentially influencing the utilization of social or health service offers (see Figure 1) indicated that the large majority of factors discussed were considered as ‘very important’ or ‘important’ in both sites, with about half of the factors receiving such a high valuation from at least nine out of ten respondents in both sites.

The uni-variate examination between select variables and ‘any social or health service’ utilization (past 30 days) in the two local study samples found the following associations. Specifically, sex (female) was significantly associated with service utilization in both sites (p < 0.039 for Rio and p < 0.041 for Salvador, respectively); furthermore, length of crack use in Rio (p < 0.018), and unstable housing status in Rio (p < 0.054) were borderline associated with service utilization. A final multivariate analysis of these factors’ associations with service utilization was precluded due to the small sample sizes and overlapping confidence intervals.

### Qualitative results

The qualitative results from the focus groups were separated into two initial categories, namely data on a) physical and mental health care, and b) drug abuse care.

#### Physical & mental health care access, barriers and needs

Almost all respondents in both sites suggested that they only seek physical health services in case of emergencies, choosing not to try and access such services for preventive or non-emergency reasons. In Salvador, most respondents reported general health care access barriers such as: long wait times; closure of service before patients are seen; lack of doctors, dentists and other professionals; lack of resources (e.g., medications). As one participant explained: *“[The] last time I went to a health services…it was very distressful, because the lines are very long…if someone needs a bandage for the foot or help to extract a tooth, there are too many lines, the person is humiliated in front of everybody and if I am under crack intoxication it is even worse, in this case I don’t wait in the line, I leave right way…”* Similarly, another participant elaborated: *“Some time ago I went [to a health service], I had dengue, with very high fever. The health service closed and I was not attended”.* [Note: All quotes have been translated from Portuguese into English by the authors].

In contrast, some respondents in Rio reported they were satisfied with the care provided by the health services they used, but complained of prejudices by health service professionals encountered by homeless patients (e.g., crack users). A participant illustrated how he experienced this: *“Hey crack man, get out! This is no emergency room!*”

Participants also cited excessive bureaucracy (e.g., requirement of proof of residence even when in need for emergency care in the public system) and high transportation costs in order to get to and access services. A few respondents in both sites stated a desire that there should be basic clinical care services, such as dental and laboratory examination (e.g., integrated in drug abuse treatment facilities). Moreover, they stated a desire for better and more psychiatric and psychological treatment services for mental health problems experienced in the context of crack dependence. One participant described: *“[…] where I live is a community health centre where I usually get condoms…to avoid the diseases. But there is no dentist. And I think that this is very wrong because all crack user need a dentist. If there is no dentist the teeth will be gone, will get rotten”.*

#### Drug abuse treatment access, barriers and needs

Virtually all individuals in both sites reported intense desires to stop using crack. One participant elaborated on their experience: *“After smoking, I get depressed, I think about my family, I am here using drugs and my kids are far way, I become regretful because I had used. Then sometimes, I stay one whole day without using, I won’t use crack anymore, I will only smoke pot and all that. But when I get money, then it is like a disease, the desire arrives immediately, the craving, then I use it again, then the sadness hits my again – shit, I had fifty Reais and I spent it all on crack, I did not even buy no food ─ then the sadness, the regrets come again, do you get it? It is like that”*. Another one emphasized what his/her desires were: *“One year from now, I intend to be free of this drug, without a trace of it…one year from now I would like to be working, to have a family, to have a good job, to be what kind of person […] to be admired by my family. What I think for one year from now is that…to be alive!”*

However, respondents demonstrated little knowledge about the availability of or access procedures to drug abuse treatment facilities, and mostly were under the impression that there is a severe lack of such services. Conversely, most participants perceived there to be several shelters available in their cities that were prepared and open to receiving crack-addicted individuals. However, they considered those shelters rather unsuited to help or support the treatment of their addiction problems, also because they were considered dirty, disorganized, without leisure activities (for distraction) or longer-term treatment resources that would enable them to abstain from crack use.

One of the participants recalled that he entered a shelter and left shortly after because *“[…] It was a pig stall! […] On the streets, at least you can look for a clean place to sleep, but in that shelter no, you have to sleep there…in that filthy place…you have to use that stinky bathroom…I don’t even want to remember that.”* Yet then, there were some discordant voices who saw good sides to the shelters: *“But the food there was good”* and *“It is better to sleep there in peace […] than to sleep in the streets doing shit”.*

One Salvador participant explained: *“… [The] drug abuse facilities do not have a television set. What they should have? A sports area, some computers for the users to learn something, to get a job, what else? A car repair shop, so that the users would leave with a job…so to be able to have good food, every day”*.

Several participants from Salvador reported ongoing active crack use within such services. Respondents in Salvador also reported expressions of prejudice by drug addiction service professionals, saying that many professionals were not skilled or qualified to treat their addiction problems.

“[…] What is missing there, it is not so much with the doctors, but with the people who work there…they were trained to do what? […] In many health centres, when the person gets there, the employee who will attend you is looking at you angry, many times turning their back on you, talking on the cell phone. So we may die there and he is talking on the cell phone, I think it is completely wrong, it is a lack of training”.

Almost all participants in Salvador suggested that effective crack addiction treatment needs to start with inpatient treatment, including access to appropriate medications to support abstinence from crack use. Numerous respondents illustrated that because of the overwhelming cravings and desires to use crack in the context of addiction, they are not successfully able to continue and complete treatment if they have easy exposure or access to environments where they usually consume or buy crack. As two participants explained: *“[…] If the person has easy access to the streets…he may relapse…so the person has to be in inpatient treatment [in order for treatment to work]”* and *“crack use has to be controlled [because] if you have access to it, you will use it”*.

Some respondents from Rio echoed these observations, although not in as large numbers as observed in Salvador. Rather, most respondents in Rio reported the desire to be able to freely access and leave services or shelters as they wished and felt necessary.

Virtually all respondents in both sites voiced a desire for availability and access to addiction services that were available 24 hours a day, 7 days a week in order to effectively address their service needs in the context of their strong and omnipresent compulsions to use crack. Given the large proportion of homeless individuals in the samples, the need for shelters exclusively for crack users was also widely voiced. As one participant stated: *“Talking about the schedule needs, there are different kinds of crack users. Some crack users are homeless, some have houses, some users go to the gutter, start to use on and on, so it is necessary to be different kinds of services…”.*

Similarly, the vast majority in both sites stated a strong need for addiction treatment services with resources and programming – including leisure, games and sports activities – to distract crack users’ minds from their ubiquitous and strong cravings to use crack. As one participant stated: *“[There should be] movies that everyone could watch …to have a course…those courses about informatics, something to occupy the mind!”*

Most respondents also suggested that addiction treatment facilities should include and offer educational or professional courses in order to help with their social reintegration and to keep them away from the dangerous lure of crack use as part of effective recovery. Two respondents explained their desires: *“[We should have a] way of making money, with something that we like to do […] and after work, to have a time of leisure, do you understand? To do things that occupy the mind!”* and *“I would like to go back to work! I would like to get my job back, to be trained […] to be able to go back to school!”*

All respondents also demanded that addiction treatment services should be staffed by professionals who are better trained, skilled, open, respectful, specialized in addiction treatment, capable of understanding the difficulties and severity of the experience of crack addiction, and sincerely willing to help them in rebuilding their lives. One Salvador participant expressed: *“[…] And doctor, if everything that I and my friends said here existed, I guarantee to you that I would sign a commitment and would enter this treatment center right now. Because I really don’t want to just be using crack. If I have a choice, I will not die because of crack. I really want to quit yet all that we just mentioned is lacking”.*

Some respondents in Rio furthermore stated that the existence of strict rules and schedules within their treatment programs would be necessary and helpful to increase the prospects of positive treatment outcomes. Some respondents voiced a desire to be able to smoke marijuana in the context of crack addiction treatment, as the use of marijuana would reduce their craving for crack and help them with their sleep and appetite.

## Discussion

This study examined patterns, determinants and unmet needs of health and social service utilization in two samples of young crack users, recruited from impoverished communities in Rio de Janeiro and Salvador, by drawing on multi-methods data. In addition to characteristics of social marginalization – as indicated by high proportions of unstable housing as well as exposure to law enforcement – our samples featured (especially considering their young age) comparably long histories of crack use, and high prevalence of use of other drugs as key risk factors for health problems [11,22,57]. This indicates both the young ages at which many crack users in Brazil begin their drug use careers, yet also reflects the realities of poly-substance use observed among many crack user populations in Brazil and elsewhere [9,55,58].

Substantive pluralities of study participants in both sites reported both compromised physical and mental health statuses and, correspondingly, high rates of physical and mental health problems; large extents of problems in both these categories were seen as linked to participants’ drug use. Irrespective of the specific nature of these health problems, these general results are in line with findings from other studies confirming the common occurrence of diverse, overlapping and commonly chronic physical and mental health problems among marginalized drug user populations, and specifically crack user populations [9,23,24,26,59,60].

In the context of high rates of physical and mental health problems in our sample as a first key finding, a second cluster of relevant findings is that our samples’ overall utilization of various social or health services was markedly low. This confirms widely observed patterns of low social or health service utilization in different jurisdictions and settings [42,61,62]. Moreover, most of the limited service utilization reported referred to social (e.g., food banks, shelters) rather than specific health or treatment services aiming at drug, or specifically crack, use problems. Moreover, the majority of respondents with physical or mental health problems did not receive medical attention for these issues, even though most expressed the need or desire for such. These low utilization patterns were categorically similar in both study sites.

These results strongly suggest a situation of substantive discrepancies or gaps between apparent physical and mental health needs, and service utilization among the two local samples of young Brazilian crack users included in this study. This situation naturally provides grounds for considerable concern, as many physical or mental health problems reported among marginalized street drug users can be chronic and/or severe, and are associated with extensive disease burden [59,63-65]. A crucial question that is then raised is what the key reasons for or barriers influencing these substantive gaps in service utilization are. Data suggest that basic levels of social and health services are, in theory, available in the contexts of both study sites, so it becomes crucial to examine and understand why these services were not better utilized by the crack user populations under study [61,66,67].

Mainly drawing on our qualitative data, our results suggest that in regards to the services available, the vast majority of participants do not see these services well-suited for their needs, and they do not see themselves well-served in these facilities. While some of these barriers appear to relate to general problems, like lack of service capacity or resources, others refer to mundanely practical or bureaucratic issues (e.g., proof of residence) that are proven to affect marginalized populations more tangibly than others. Notably, participants also emphasized that they perceive many service providers not to be adequately prepared, qualified or experienced to deal with addiction-related health problems [68], or simply exert active stigma or prejudice against crack users preventing them from better utilizing much needed services. These obstacles and barriers to services targeting especially marginalized drug users specifically are well documented in other jurisdictions, and recognized as major contributors to highly compromised health status and care in these high-risk populations [42,67,69,70]. Furthermore, our exploratory quantitative analyses of factors univariately associated with service utilization suggested that sex, and possibly housing status specifically appear to further influence the likelihood of service utilization in both sites.

In Brazil, health and drug abuse treatment services are offered and organized in different ‘networks’ of services, including institutionalized (e.g., hospital-based) and community-based care (e.g., community-health centres; [71]). Most of the institutionalized care is accessed only in the rarest of circumstances by marginalized drug users, due to their ‘high-threshold’ design and operations. It has been recognized that vastly more community-based services are most urgently needed for social and health service provision for marginalized drug users, yet despite the recent expansions of ‘CAPS’ services as the core strategy across Brazil, there are several systemic and structural barriers towards better utilization and effectiveness [38,39]. For example, there are much fewer CAPS in number than what has been estimated to be needed for effective service delivery. In Rio de Janeiro, a minimum number of 30 CAPS-AD had been projected for adequate service delivery, yet only 3 (i.e., 10%) existed at the time of the study [72]. In addition, public care services are vastly under-resourced (leading to programming and service restrictions as well as overload) and facing severe staffing problems, including inadequately trained and/or un-motivated staff, and high rates of staff loss and/or turnover. These problems are amplified by the competition of the expanding and booming private health care sector in Brazil, which is commonly ‘creaming off’ the best professionals with much higher pay and better working conditions [73,74]. It is reasonable to assume that these – commonly experienced and real – negative conditions together are keeping marginalized drug users from more frequent service utilization. Hence, one part of necessary remedies certainly is a substantive expansion of services in quantity and availability. A second part, however, is a substantive improvement in service resources and service quality, especially targeted to the distinct needs of marginalized drug users, as specifically documented by our study’s data. Other systems have responded to similar circumstances by establishing both ‘low threshold’ health and social care offers to marginalized drug users that provide services explicitly and most essentially desired by the target populations (e.g., basic food, shelter and hygiene services, flexible opening hours, social and educational supports, health and treatment care referrals etc. [54,75-77]). These have generally led to improved basic service utilization as well as improved linkages and referrals to institutionalized care (e.g., mental health, drug abuse treatment), and hence may offer exemplary models for concrete community-based service expansion and improvements in Brazil. Our findings further confirm recent data from Brazil and other jurisdictions that socially distinct sub-populations face even more pronounced barriers to service utilization than drug users in general [52,78-80], and require special attention, confirming well-documented ‘social determinants’ of health or health care access even within contexts of marginalized drug users [81,82]. Brazilian policy documents [83] recognize the major gaps in adequate (e.g., primary) health care especially for marginalized populations, yet remedial measures are far from sufficient and ought to be vastly expanded and accelerated.

Complementing our study’s results indicating substantive service access and utilization gaps among crack users, it offers important evidence on the study population’s acute needs in these realms. Concretely, substantive majorities of participants in both sites reported that they, if available, would utilize basic social or health services specifically designed and tailored for drug users. Details of our quantitative data list a number of key components – ranging from both basic existential (e.g., food and hygiene) and social support services to specialized health and addiction care services, yet also access and control features – that are considered important to make such facilities appealing and attractive for utilization see Figure 1. These mentions underscore the study populations’ needs for basic and existential social and health care that have also been reported for similar populations in other contexts [12,34,42,84-87]. Other Brazilian studies [88] have underscored the importance of improved social and professional supports and reintegration as a key factor for effective treatment strategies for crack users.

Notably, our qualitative data especially emphasizes a strong need and desire for adequate addiction treatment or care services among the study participants that would allow them to effectively address and treat their substance use problems, primarily crack addiction. While it is important to recognize that desires for addiction treatment or care are commonly present yet also differ in specifics in this population, it is principally disconcerting that most users currently do not see adequate treatment service or options available to them. As noted elsewhere, crack abuse or dependence is a form of psychoactive drug use for which few standard and commonly available treatment interventions exist [89-91]. However, even on this basis, little to none appear to be available to study participants in their respective Brazilian contexts.

This study has some important limitations. It is based on local convenience samples, which may involve participation bias, and not be representative of crack user populations in the study sites, or elsewhere in Brazil. Data are based on self-report, which may influence validity, although great care was exercised (e.g., by protecting participants’ anonymity, using skilled interviewers trusted in the study communities) to address key sensitivities; validity in studies relying in similar approaches has been found to be good [92].

## Conclusions

Based on the above, our study provides major implications for policy and practice as related to social and health service development and implementation for the large populations of crack users in the two Brazilian study sites (and likely reflect similar situations in other Brazilian cities). While our study samples were characterized by high levels of physical and mental health problems, and substance use co-morbidities, current health and social services are largely perceived to be inaccessible, inadequate or unappealing; in addition, special interventions designed to meet crack users’ service needs – also concerning addiction treatment – largely appear to not exist. Given the high prevalence of and extensive health problem burden related to crack use in Brazil, these service gaps urgently need to be ameliorated with targeted and effective measures. This will need to include tearing down and addressing key barriers and obstacles to utilization of existing services, yet also the development and implementation of new and better social, health and treatment service options for the target population.

## Appendix 1

### Focus Group Question Guide (translated from Portuguese by the authors)

1. Which kind of health facilities have you used recently? Tell us about the last time you went to a health service. What was your experience like?

2. Have you had any difficulties in the last service you used? If yes, which difficulties? In case there were no difficulties with your last visit, have you ever had any difficulty when using a health service?

3. Which services for drug users would you like to use and how do you think those services should be designed/what should they offer?

4. How do you think that, for yourself, good addiction treatment should be offered and done? For example, which services and activities should be included? Who should provide the treatment? Where should it located?

5. What would be the best access and service arrangements (e.g., re: opening hours)? How would you like to use the service, e.g. how many times a week, which days/hours?

6. In other interviews, crack users told us that drug abuse treatment facilities should provide referral to shelters. How should such shelters be designed and operated in order to work for you? Have you ever been in a shelter? What was your experience like?

## Competing interests

The authors declare that they have no competing interests.

## Authors’ contributions

MC, TA, EL, FB, BF designed and co-led the study and the analysis plan. NB, LL and CB co-executed literature reviews and assisted with quantitative and qualitative data analyses. BF led the write-up; all authors reviewed and substantively contributed to draft revisions, and approved the draft as submitted. All authors read and approved the final manuscript.

## Pre-publication history

The pre-publication history for this paper can be accessed here:

http://www.biomedcentral.com/1472-6963/13/536/prepub
